# Fully Aqueous Self-Assembly of a Gold-Nanoparticle-Based Pathogen Sensor

**DOI:** 10.3390/ijms24087599

**Published:** 2023-04-20

**Authors:** Timothy Robson, Deepan S. H. Shah, Rebecca J. L. Welbourn, Sion R. Phillips, Luke A. Clifton, Jeremy H. Lakey

**Affiliations:** 1 Biosciences Institute, The Medical School, Newcastle University, Framlington Place, Newcastle upon Tyne NE2 4HH, UK; 2 Orla Protein Technologies Ltd., Biosciences Centre, International Centre for Life, Times Square, Newcastle upon Tyne NE1 4EP, UK; 3 ISIS Pulsed Neutron and Muon Source, Science and Technology Facilities Council, Rutherford Appleton Laboratory, Harwell Science and Innovation Campus, Didcot, Oxfordshire OX11 OQX, UK; becky.welbourn@stfc.ac.uk (R.J.L.W.);

**Keywords:** outer membrane protein, scFV, LSPR, gold nanoparticle, influenza, nucleoprotein, protein engineering, silanization, AAPTMS, PEDA

## Abstract

Surface plasmon resonance (SPR) is a very sensitive measure of biomolecular interactions but is generally too expensive for routine analysis of clinical samples. Here we demonstrate the simplified formation of virus-detecting gold nanoparticle (AuNP) assemblies on glass using only aqueous buffers at room temperature. The AuNP assembled on silanized glass and displayed a distinctive absorbance peak due to the localized SPR (LSPR) response of the AuNPs. Next, assembly of a protein engineering scaffold was followed using LSPR and a sensitive neutron reflectometry approach, which measured the formation and structure of the biological layer on the spherical AuNP. Finally, the assembly and function of an artificial flu sensor layer consisting of an in vitro-selected single-chain antibody (scFv)-membrane protein fusion was followed using the LSPR response of AuNPs within glass capillaries. In vitro selection avoids the need for separate animal-derived antibodies and allows for the rapid production of low-cost sensor proteins. This work demonstrates a simple approach to forming oriented arrays of protein sensors on nanostructured surfaces that uses (i) an easily assembled AuNP silane layer, (ii) self-assembly of an oriented protein layer on AuNPs, and (iii) simple highly specific artificial receptor proteins.

## 1. Introduction

The use of periodic, nanostructured metallic arrays for sensing applications has been widely investigated [1]. By far, the most studied metals are gold and silver, which display useful optical phenomena that are sensitive to the local environment [2]. These phenomena are due to the coherent excitation of conductance band electrons by electromagnetic radiation, known as surface plasmon resonance (SPR), which is also the basis of the sensing capabilities of thin planar gold films [3]. The resonance response can be tuned by changing the size and periodicity of the nanostructures. A wide range of differently shaped nanostructures have been fabricated using various techniques, the most versatile of which are the lithographic methods. Arrays of spheres, disks, holes and triangles have been made using nanoimprint [4], nanosphere [5] and focused ion beam lithography [6]. Whilst able to generate precisely structured arrays, lithographic techniques can be time-consuming, costly and require specialized equipment.

Alternatively, surfaces can be decorated with assemblies of nanoparticles by deposition from solution [7,8,9], a process that is relatively straightforward and can be carried out on the bench. To encourage nanoparticle binding, the surface is usually pre-functionalized with a chemical group such as a primary amine. A range of different nanoparticles have been assembled on various surfaces, with the most widely studied being gold nanoparticles (AuNPs) on functionalized glass [8,10]. Although these assemblies do not have the precisely ordered structure of lithographic arrays, they still present tunable SPR behaviour [3] that can be used for sensing applications [7,11]. In addition to its low cost, glass is an attractive substrate because it can be made into a variety of different shapes, easily integrated into optical systems and functionalized with a wide range of silane-based surface chemistries.

Protein assemblies on both nanoparticle and planar biosensor surfaces are routinely used to provide specific detection of a particular antigen or protein, with antibodies being the most used [12]. While it has been shown that control over the orientation and structure of these protein layers improves the functionality of the resulting sensor surface [12,13], there are relatively few studies that have investigated the protein layer structure. The oriented assembly of engineered membrane protein arrays on planar gold surfaces has previously been shown using neutron reflectometry experiments [14,15], but studies of more complex nanostructured surfaces have not been carried out. Here we present a simple method for the generation of AuNP assemblies on aminosilane functionalized glass surfaces where all of the steps are carried out in aqueous solution at room temperature. Furthermore, we use neutron reflectometry to show that an engineered protein scaffold assembles in an oriented fashion on the surface of the deposited gold nanoparticles, thus providing a high degree of biological functionality to the glass surface [16]. The assembly and functionality of these protein-nanoparticle surfaces was then confirmed via an LSPR assay using 20 nm AuNPs deposited on borosilicate glass capillaries. This measured the binding of a clinically relevant antigen, influenza A nucleoprotein (FluA NP) [14,17], to a scaffold protein engineered to contain a single-chain antibody domain.

## 2. Results

### 2.1. Deposition of Gold Nanoparticles on Aminosilane Functionalized Glass Surfaces

**Figure 1:**
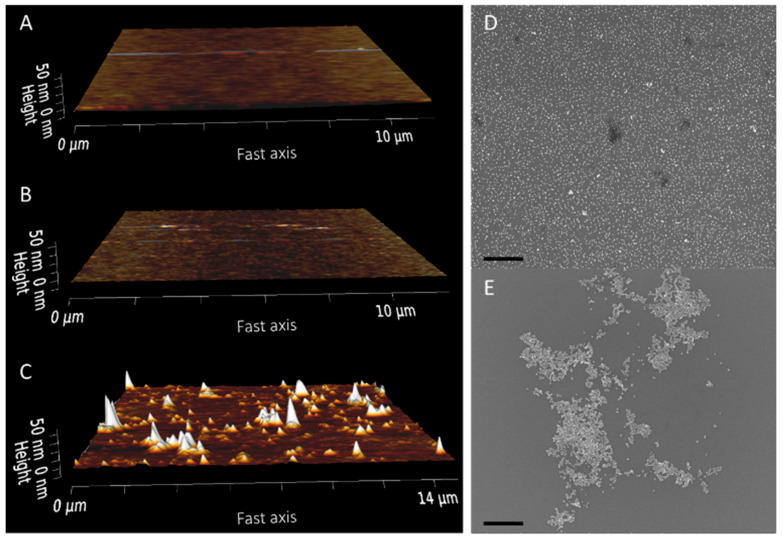
Aminosilane functionalization of silicon wafer segments and subsequent gold nanoparticle deposition. AFM images of a blank wafer (**A**) and wafer segments after AAPTMS (**B**) and APS (**C**) functionalization. Representative SEM images of AAPTMS (**D**) and APS (**E**) functionalized surfaces after deposition of 10 nm gold nanoparticles. Scale bar = 500 nm.

Glass surfaces were first prepared with an aminosilane—(aminoethylaminomethyl) phenethyltrimethoxysilane (AAPTMS or PEDA [18])—to form an adhesion layer for the deposited gold nanoparticles. This silane was chosen as it generates smooth monolayer surfaces when deposited from an aqueous ethanol solution, as shown by AFM (Figure 1B), and did not require subsequent annealing steps [19]. The structure of AAPTMS monolayers has not been rigorously investigated; however, Chen et al. [20] have proposed a model for cured AAPTMS monolayers where the structure is reinforced by hydrogen bonding between the terminal amine groups and hydrophobic interactions between the aryl groups, such as π-stacking and van der Waals forces. This is consistent with the organization observed for other aryl-containing silane molecules [21] and would suggest that the introduction of aryl groups could be used to encourage orientation of the silane monolayers. By comparison, surfaces of (3-aminopropyl)trimethoxysilane (APS)—a very commonly used silane for the formation of amine surfaces on glass substrates [8,10,11]—generated using the same aqueous method presented large aggregate structures when imaged (Figure 1C).

**Figure 2:**
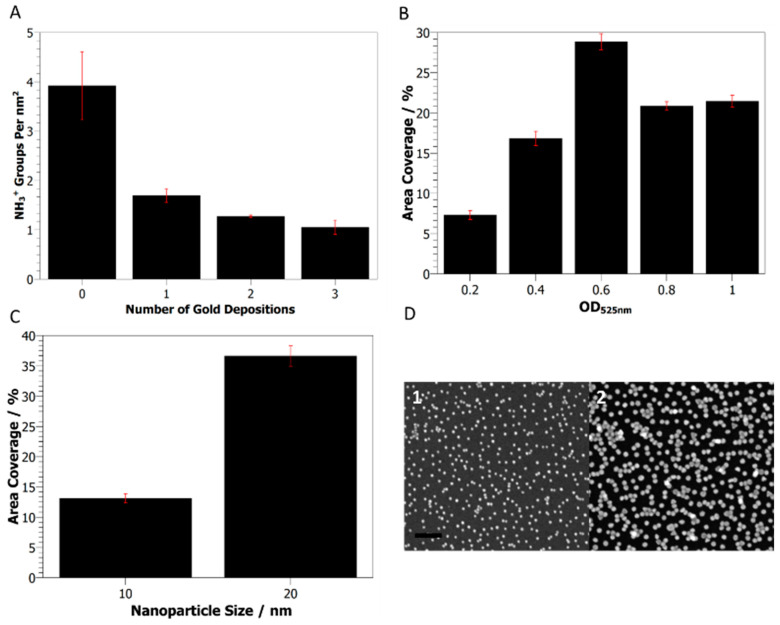
Gold nanoparticle deposition on AAPTMS functionalized silicon wafers. (**A**) The density of primary amine moieties on the wafer surface after multiple 10 nm gold nanoparticle depositions. (**B**) Area coverage of 10 nm gold nanoparticles on the surface after deposition with increasing nanoparticle concentration. (**C**) Effect of nanoparticle size on surface coverage. (**D**) Representative SEM images of deposited 10 nm (1) and 20 nm (2) gold nanoparticle surfaces. Scale bar = 100 nm.

The formation of a smooth aminosilane monolayer was shown to be critical for depositing even fields of nanoparticles on the surface, as shown by SEM images of the AAPTMS sample. By comparison, the APS sample showed large, aggregated clusters of particles when imaged by SEM (Figure 1D,E). AuNP binding to aminosilane surfaces is primarily mediated through charge interactions between the positively charged primary amine group and negatively charged ligands on the nanoparticle surface [7,22]. Analysis of the available amine density of AAPTMS surfaces, using a colorimetric Coomassie assay [23], showed a continued reduction in the NH3+ density after multiple gold depositions (Figure 2A).

Modulation of the AuNP coverage was possible by varying the concentration of the nanoparticle solution or changing the particle size (Figure 2B,C). Increased surface coverage was observed by SEM when increasing either the nanoparticle concentration, from an OD525nm = 0.2–1, or particle size, from 10 to 20 nm. Above 0.6 OD, 20 nm particles appear to be saturated on the surface (Figure 2B), and the number of immobilized AuNPs does not scale with particle number in solution since there are approximately eight times as many 10 nm particles as 20 nm particles per mL of coating solution at 1 OD525nm but similar numbers on the surface (Figure 2D).

### 2.2. Self-Assembly of Engineered Protein Layers on the Surface of Deposited AuNPs

Protein assembly on the nanoparticle surface was investigated with a protein scaffold that uses the self-assembly properties of an engineered bacterial membrane protein (OmpATM) to coat gold surfaces [14,24]. The protein attaches via an inserted cysteine residue that provides gold–sulfur bonding. Here we used the protein chimera GGzOmpATM, which combines tandem B-domains from protein G (GG) [25] and a Z-domain from protein A (z) [26] bound to the N-terminus of a circularly permuted OmpATM [14,27]. Both B and Z domains bind to the constant (Fc) domain of immunoglobulin G (IgG) antibodies, enabling their oriented presentation on surfaces [15]. An established assembly method, developed for planar gold surfaces [28], was used for AuNPs that had been deposited on silane-modified single-crystal silicon blocks and surface passivated with β-mercaptoethanol [29]. Incubation with GGzOmpATM was followed by the addition of a lipid-mimicking molecule, 1-mercaptoundecyl-11-hexaethylene glycol (filler). Co-assembly of the filler molecules on the protein-coated surface helps to stabilize the membrane protein array, encourage orientation and reduce non-specific binding [14,28]. Poorly bound protein and filler molecules were removed from the surface by washing with 1% SDS, followed by HEPES buffer to remove SDS.

While nanoparticle assembly on the surface can be easily imaged by electron microscopy (Figure 2D), the structure of the assembled protein array on the AuNP surface presented a more complex challenge. To address this problem, we used specular neutron reflectometry (NR) to investigate the protein-coated AuNP surface. This technique can measure the interfacial composition perpendicular to the solid-liquid interface (termed the z-axis) by reporting differences in the neutron scattering length density (nSLD), which can be thought of as a neutron refractive index. NR is particularly sensitive to light elements and hydrogenous materials, making it ideal for the interrogation of protein layers in complex systems [15,30]. Furthermore, ^1^H and ^2^H (D) display very different nSLD; thus, isotopic exchange of the background aqueous phase can be used to highlight different regions of the interfacial structure. By matching the nSLD of the water subphase to specific components of the complex surface, we can render these invisible and highlight the spatial distribution of the remaining components. Taking multiple datasets of the same surface while exchanging the water subphase with different ratios of H:D then allows the surface composition and structure to be extracted. Reflectometry data were collected for the AuNP surface before and after protein assembly. Measurements of the AuNP surface were carried out in three water subphases; H_2_O, D_2_O and a mixture of these with nSLD matched to silicon—silicon matched water (SMW). This simplifies the data since matching silicon and water “refractive indices” reduces the large signal from silicon interface reflection. A fourth subphase, matched in nSLD to gold (gold matched water or AuMW), was also used when measuring the protein-coated surface; this removed the strong gold reflection signal, enabling the data from the biological layer to be more pronounced. The fitting models for reflectometry data are usually formed from a series of layers at the solid–liquid interface, which have individual thickness, roughness and nSLD parameters [28]. The nSLD of each layer can be calculated as a scaled sum of the constituent materials of the layer and their volume fraction. This includes any contribution of the liquid subphase to the nSLD in the outer layers (hydration), and any exchangeable protons are considered to match the H/D ratio of the bulk solution. Fitting of the spherical nanoparticles at the surface required careful construction of the model layers. Two models were considered for fitting the data in this work: a simple layer model (slab model) and a more complex spherical model (sphere model). For both models, the silicon substrate, SiO_2_ and silane layers were modelled in the same way (one slab for each), differing only in the outer layers that define the nanoparticles and assembled proteins.

The simple slab model mirrored the nanoparticle across the midline, with each half comprising five layers of identical thickness. The spherical shape was described by the layers having an asymmetric gold composition, with the gold contribution greatest at the center of the particle, and the interfacial roughness of the layers allowed the creation of a smooth rather than stepped shape. Protein assembly was modelled as a single outer coating of the AuNP, which comprised both protein and filler molecules contributing to the nSLD.

**Figure 3:**
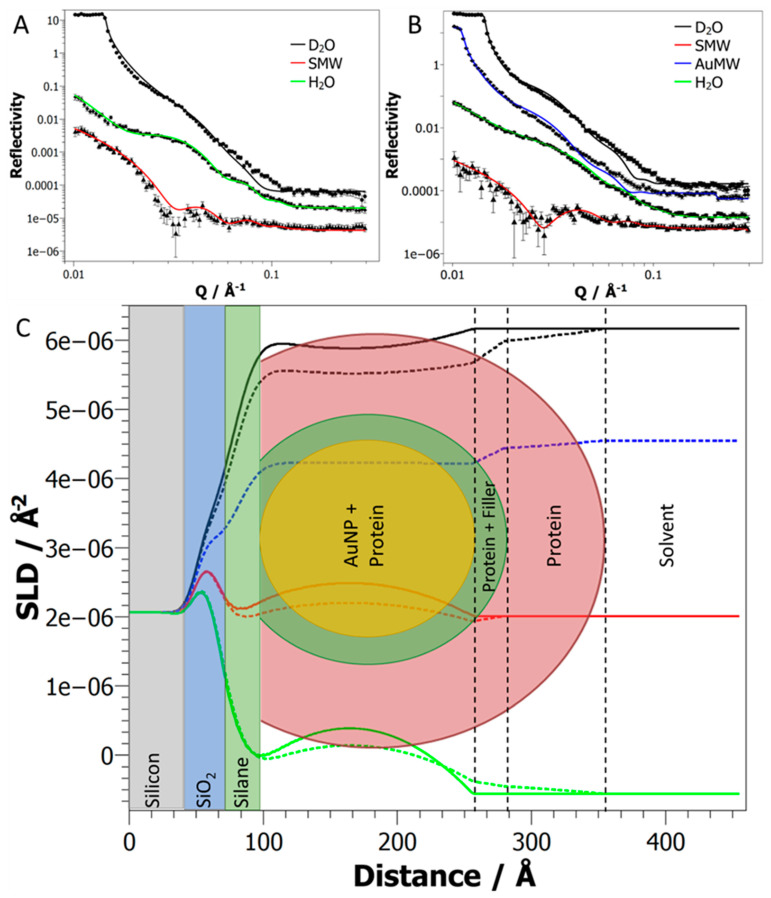
Data fitting and corresponding SLD profiles generated using the sphere model for the AuNP surface before and after GGzOmpATM assembly. (**A**,**B**) Reflectivity data for AuNP and AuNP + GGzOmpATM surfaces, respectively, with the model fits represented by solid lines and the reflectivity profiles offset for clarity. (**C**) The resulting SLD profiles from the fits of the reflectivity data. Both of the SLD profiles have been overlaid with the solid and dashed lines representing the AuNP and AuNP + GGzOmpATM surfaces, respectively. The colors represent the different solvent contrasts used; D_2_O, SMW, AuMW and H_2_O. A schematic representation of the layer structure is also overlaid, which shows the composition of the different regions of the SLD profile. **NB** 1e-06 has been used to indicate 1 × 10^−6^.

By contrast, the sphere model used a geometric description of a perfect sphere for the AuNP resting on the silane surface. The sphere was split into a series of very thin layers with a single thickness value and sphere diameter. Protein assembly was modelled as a two-part coating around the AuNP, where the inner part contained both the protein and filler molecules and the outer part contained the extended protein domains. The nSLD for each thin layer was constrained by the fractions of AuNP, protein, filler and solvent at the particular distance from the silane surface (Z), with the AuNP included as a disk with diameter given by the chord of the AuNP in the plane parallel to the surface at distance Z. For determining surface coverage, each of the spheres can be considered to reside in a box with the dimensions determined by the total thickness in each direction, i.e., the AuNP diameter plus the protein thickness in the perpendicular plane and the AuNP diameter plus twice the protein thickness in the horizontal plane. Deviation from a close-packed arrangement of the cuboids was accounted for by a coverage parameter that filled the remaining volume with bulk solvent (Figure 3).

In each model, to fit the AuNP sample, the diameter of the particles and the surface coverage were allowed to vary during fitting. After protein assembly, the thickness, hydration and coverage (over the AuNP surface) of both the inner and outer protein coatings were also allowed to vary. The nSLD of the inner layer was allowed to vary between pure thiol and pure protein (0.216 and 1.993 × 10^−6^ Å^−2^, respectively). AuNP coverages were found to be similar before (23.01 ± 6.18%) and after (23.71 ± 11.71%) protein assembly (Supplementary Tables S1 and S2); thus, the protein addition and washing steps appeared not to remove AuNPs from the surface. This was unexpected due to the number of washing steps used during protein assembly and the non-covalent nature of the AuNP association with the surface. Samples were recovered after neutron reflection experiments, and SEM imaging also showed similar AuNP coverage to that observed previously (Supplementary Information Figure S1).

The AuNP diameter was well defined by both models before protein assembly, at 179 ± 6 and 184 ± 5 Å for the slab and sphere models, respectively. However, the results of the protein coating fitting were quite different between the two models. Fitting of the slab model to the reflectivity data after protein assembly resulted in a thin protein coating, 30 ± 10 Å, and a lower particle diameter of 154 ± 14 Å, which are both significantly lower than expected (Figures S2 and S3, Tables S1 and S2). The AuNP roughness parameter that was used to create a smooth spherical shape also doubled from 35 ± 11 to 66 ± 16 Å. By contrast, the spherical model, which simultaneously fitted the datasets before and after protein assembly, described a dense inner protein coating part 23 ± 3 Å thick—covering 99.10% of the surface—and a more diffuse outer part 75.09 ± 0.18 Å thick, with a 15.62% coverage (Figure 3C, Table S3). The spherical model results were consistent with the layer structure described in previous studies using the same protein on planar gold surfaces and AuNPs in solution [15,16].The total protein layer thickness (98 ± 3 Å) was less than the 135 Å previously reported on planar surfaces, and this might be explained by particle packing. The spherical model used an ideal geometric interpretation where all of the particles were perfect spheres that packed together in an ordered fashion on the surface, remaining far enough away that the proteins could be fully extended. Some of the particles were closely associated, as observed by EM (Figure 2D), which would restrict the orientation of any bound proteins on the particles. However, the total layer thickness was significantly greater than the height of the OmpATM barrel [27] (57 Å), which suggests that a large proportion of the proteins were organized in an oriented fashion on the nanoparticle surface. Hence, while the simple slab model could robustly show that the AuNPs were the correct size and on the surface, the spherical model was able to provide further detailed information on the protein layer.

The goodness of fit of the slab model approach was generally better than that of the spherical nanoparticle model approach. This is likely due to the ability of the simple slab model approach to capture minor surface structural heterogeneities, which was not possible with the defined homogeneous spherical model. The spherical model, however, provided a quantitative assessment of the complex interfacial structure, and this was, therefore, better suited to resolving the gold/OmpA nanoparticles on the Si surface.

The data fitting and nSLD profiles generated from fitting the slab model and all the fitted parameters for both models are provided in the Supplementary Information. A similar modelling method has recently been successfully applied to NR data from surface-bound silicon nanospheres (50–200 nm) coated with lipid bilayers. Here, the spheres are modelled as 2000 slices, each formed of concentric cylinders describing the different nSLD of the components [31].

### 2.3. LSPR Detection of Protein Assembly and Antigen Binding

**Figure 4:**
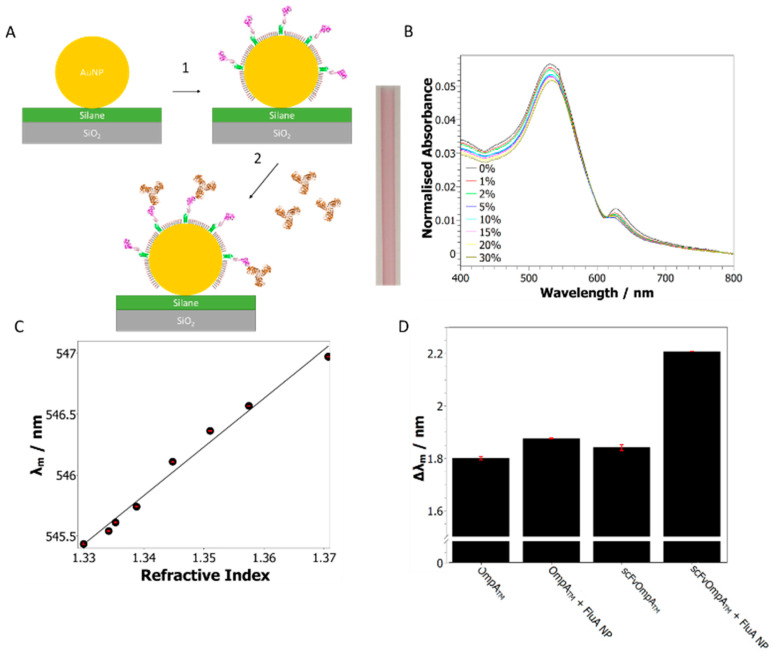
Detection of influenza A nucleoprotein using scFvOmpATM functionalized gold–glass capillaries. (**A**) Schematic diagram of scFvOmpATM assembly on the surface of gold nanoparticles deposited on a glass capillary, showing the OmpATM scaffold bound to the gold and the scFv domain displayed away from the surface (1). The scFv domain is then free to bind FluA NP antigen that is in solution (2). The image in the center shows a AuNP functionalized glass capillary with its uniform red color. (**B**) UV-Vis spectra of AuNP-coated capillaries with solutions of increasing glycerol concentration. (**C**) Analysis of the UV-Vis spectra showing the linear relationship between the mean wavelength (λm) and refractive index for the glycerol stock solutions. This revealed a sensitivity of 39.95 ± 0.02 nm/RI unit. (**D**) In situ assembly of scFvOmpATM and OmpATM control proteins and subsequent injection of 60 µg/mL FluA NP solution as followed by UV-Vis spectroscopy. Assembly of both proteins can clearly be seen as a shift in the λm; introduction of FluA NP into the capillary caused a larger shift in the λm for the scFvOmpATM surface than the OmpATM control (0.367 ± 0.01 and 0.076 ± 0.006 nm, respectively).

An LSPR assay was developed to confirm that the assembled AuNP–protein arrays, based on OmpATM, retained their biological activity. This assay also demonstrated the utility of this system for the simple generation of sensor substrates that can be analyzed using standard benchtop equipment. Firstly, the interiors of borosilicate glass capillaries were functionalized with 20 nm AuNPs as described in Methods. The functionalized capillaries were then used as a convenient flow cell that could be mounted in a standard UV-Vis spectrometer. The functionalized capillaries were a uniform red color and presented a localized surface plasmon resonance (LSPR) peak with a maximum wavelength of 535.6 nm when measured in water (Figure 4B). The sensitivity of the LSPR peak to changes in the refractive index was measured using the shift of the barycentric mean wavelength λm (see Methods) when exposed to glycerol stock solutions of increasing concentration, from 0–30% *w*/*w*. This revealed a sensitivity of 39.95 ± 0.02 nm/RI unit, which is comparable to previous studies on AuNP-coated glass surfaces [7,19] (Figure 4B,C).

A chimeric OmpATM protein was assembled on the AuNPs to generate a specific sensor surface that could bind a defined protein antigen. Specific binding was facilitated by using a bespoke single-chain antibody variable domain fragment (scFv) fused to the OmpA scaffold through an alpha-helical linker region (scFvOmpATM) [16]. The scFv domain used in this study was selected to bind influenza A nucleoprotein (FluA NP), an RNA-binding protein from the influenza virus, via a bacterial retained display platform and affinity selection techniques [32]. These antigen-specific scFvOmpATM proteins can then be made in large quantities by bacterial fermentation, avoiding the need for animal-derived antibodies, and can be assembled in one step, as shown here and previously [16]. Influenza nucleoproteins are the preferred target for influenza detection, as they are highly conserved and type specific [14,17]. Assembly of scFvOmpATM and an OmpATM -only control protein was carried out in situ on separate glass capillaries and could be observed by a shift in the λm, when compared with bare nanoparticles, of 1.840 ± 0.01 and 1.799 ± 0.006 nm, respectively (Figure 4D). After assembly, a 60 µg/mL solution of FluA NP was injected through the capillaries before measuring the UV-Vis spectrum again. A much greater shift in the λm was observed for the scFvOmpATM surface than the OmpATM control (0.367 ± 0.01 and 0.076 ± 0.006 nm, respectively), indicating that specific binding occurred between the scFv domain and FluA NP. This wavelength-based approach differs from the original capillary-based LSPR sensors that relied upon absorbance changes [33], which are easier to measure but dependent upon the relative value of the intensity. The barycentric mean analysis could enhance the accuracy of wavelength-dependent capillary sensors [19]. The simpler NP and protein immobilization methods are, however, applicable to any mode of analysis.

## 3. Discussion

There has been much work published in the field of LSPR sensors, and the recent review by Masson [3] provides an important update. Compared to gold films, AuNP-based devices have lower sensitivity but offer ease of formation and use [7,34]. One pertinent example is the assembly on AuNP of aptamers able to detect Salmonella bacteria and their coating of transparent surfaces for quality control of pork meat [35]. Interestingly, this study used a normal silane linker assembly at 50 °C for 1 h, compared to our ambient assembly. Against this rich background, the work presented here reports methods to simplify the layer-by-layer process and demonstrates that AuNPs deposited onto smooth aminosilane monolayers provide a stable platform for the self-assembly of arrays of engineered sensor proteins on glass and silicon surfaces. The spacing of the AuNPs observed by TEM showed a high efficiency of assembly that approaches that obtained from more complex manufacturing systems, such as soft lithography [36] or ion beam irradiation [37]. It does not recreate the regular spacing that is required for stronger plasmonic effects, but the LPSR signals obtained are easily measured by a simple laboratory spectrometer. Having achieved this high density of AuNPs, the subsequent in situ assembly of the sensing protein layer was a new challenge, in particular, the need to avoid removal of AuNP from the surface. This was achieved, and TEM showed unchanged AuNP data even after lengthy NR experiments. The use of NR to study layer structure on arrays of nanoparticles [38] presents challenges in analysis that are only recently being solved [31]. Here, the two approaches revealed a similar layer structure to that observed in previous studies on gold films [15] and AuNPs in solution [16], with the proteins assembled in an oriented fashion and displaying their functional domains away from the AuNP surface. Glass surfaces functionalized with 20 nm AuNPs using this method exhibited a clear LSPR response and were sensitive to changes in the local refractive index. However, it is likely that the sensitivity would need to be improved before practical application [3]. The wavelength shifts are smaller than with patterned surfaces [37], but the barycentric wavelength analysis provides a robust measure of these small shifts. Assembly of an OmpATM protein engineered with a single-chain antibody fragment—scFvOmpATM—could be observed by a shift in the LSPR peak as well as subsequent specific binding of a clinical antigen—FluA NP—that is routinely used in the diagnosis of influenza infections. Using aqueous conditions friendly to biological components, this work provides direct evidence for sequential assembly of the silane, AuNP and oriented engineered protein layers. These have the potential to be used as versatile, cost-effective sensor substrates that can be analyzed with commonly used UV-Vis spectrometers.

## 4. Materials and Methods

Materials. Gold nanoparticles were purchased from BBI Solutions (Cardiff, UK). AAPTMS was purchased from Fluorochem Ltd. (Hadfield, UK). Molecular biology and protein purification materials were purchased from Invitrogen, Generon and GE Healthcare (Amersham, UK). All other materials were purchased from Sigma-Aldrich (St. Louis, MO, USA) unless otherwise stated.

Protein Production and Purification. GGzOmpATM and scFvOmpATM were expressed, purified and refolded as described previously for GGzOmpATM [14]. The bespoke scFv domain was purchased from Affinity Bio (Scoresby, Australia) [32]. Recombinant influenza A nucleoprotein expression and purification were carried out as described previously [14,17].

Silanization. Silicon wafer chips (Agar Scientific, Stansted, UK), approximately 1 cm × 1 cm in size, were cleaned using Piranha solution (3:1 sulfuric acid: hydrogen peroxide (30%)) before being washed with copious amounts of Nanopure water. 1 mL of silane solution was added to 94 mL of ethanol and 5 mL of water and stirred for 10 min to activate the silane. The activated solution was then diluted 1:2 in ethanol before use. Piranha-cleaned wafer chips were suspended in the silane solution using a stainless steel rack and incubated for 30 min with stirring. Functionalized wafer chips were washed with ethanol and sonicated three times for 1 min in an XB2 ultrasonic bath (Grant Instruments, Cambridge, UK). A final wash with Nanopure water was carried out before drying with an air line.

Gold nanoparticle deposition for SEM. Silanized silicon wafer chips were separated into individual wells in a 12-well polystyrene plate. 400 µL of nanoparticle solution was added to each well to cover the surface of the sample and incubated for 60 min on a 3D rocking platform (Stuart Scientific, St. Neots, UK). The samples were then washed with Nanopure water and sonicated three times for 1 min in an XB2 ultrasonic bath (Grant Instruments, Royston, UK) before drying with an air line.

Colorimetric amine density assay. A modified method from Coussot et al. was used [23,39]. Briefly, functionalized wafer chips were transferred to a 24-well plate, and 150 µL of staining buffer (0.5 mg/mL Coomassie Brilliant Blue G250, 10% *v*/*v* methanol, 5% *v*/*v* glacial acetic acid) was carefully added to the polished surface before incubating for 5 min on a 3D rocking platform (Stuart Scientific, St. Neots, UK). The staining buffer was then removed, and each chip washed 5 times with 300 µL of washing buffer (10% *v*/*v* methanol, 5% *v*/*v* glacial acetic acid) to remove unbound Coomassie. The chips were moved to a clean 24-well plate before adding 150 µL of destain buffer (50% *v*/*v* methanol, 50% *v*/*v* 0.25 M Na_2_CO_3_) and incubated for 1 min to extract the bound Coomassie dye. The destain buffer was carefully removed and added to an Eppendorf-type tube containing 3 µL of 6.85 M HCl to acidify the dye and maximize absorbance. Absorbance at 610 nm was measured in triplicate using the NanoDropTM ND1000 spectrometer (Thermo Fisher Scientific, Horsham, UK) and the Coomassie concentration calculated using the extinction coefficient of 83,100 M^−1^ cm^−1^. Coomassie binds to protonated amines with a 1:1 stoichiometry, so the amine density was calculated by dividing the surface area by the number of extracted Coomassie molecules in solution.

The amine density of triplicate AAPTMS functionalized silicon wafer chips, made as described above, was measured before incubating with 10 nm gold nanoparticles at a concentration of OD525nm = 0.3 for 10 min. The amine density was measured again, and the AuNP incubation repeated three times.

Atomic Force Microscopy. Images were acquired using a NanoWizard III Bio AFM (JPK, Berlin, Germany) on a Halcyonics Micro 40 anti-vibration table with silicon nitride 0.6 µm cantilevers (Bruker model DNP-10). Silicon wafer chip samples were mounted on a glass slide with superglue before use. The resonant frequency of each probe was measured using the built-in software before imaging. Images were collected in tapping mode and analyzed using the built-in JPK software.

Scanning Electron Microscopy. Silicon wafer chips of approximately 1 cm × 1 cm in size were functionalized with either commercial or synthesized gold nanoparticles before mounting on aluminum specimen stubs with carbon adhesive discs. Samples were visualized using an FEI Helios Nanolab 600 microscope equipped with an Elstar UHR immersion lens. Images were acquired using working distances between 3.0 and 4.5 mm and an accelerating voltage of 5 kV. Magnifications between 30,000× and 180,000× were routinely used. The resulting high-resolution images were saved as TIFs before analysis using Image J. Gold nanoparticle surface area coverage was calculated by converting the image to an 8-bit grey scale and selecting the region of the grey scale that included only the nanoparticles using the threshold tool. The % area of the image covered by the nanoparticles was then calculated using the measure tool. Three separate images were used to calculate the average surface coverage for each sample.

Neutron Reflectometry. Specular neutron reflectometry (NR) measurements were carried out using the white beam INTER reflectometer [40] at the Rutherford Appleton Laboratory (Oxfordshire, UK), using neutron wavelengths from 1.5 to 16 Å. The reflected intensity was measured at two glancing angles of 0.7° and 2.3° as a function of the momentum transfer, Qz (Qz = (4π sin θ)/λ, where λ is wavelength and θ is the incident angle). Data were collected at a resolution (dQ/Q) of 3.5% and a total illuminated length of 60 mm.

Purpose-built liquid flow cells for analysis of the silicon–liquid interface were placed on a variable-angle sample stage in the NR instrument, and the inlet to the liquid cell was connected to a liquid chromatography pump (L7100 HPLC pump, Merck, Hitachi, Tokio, Japan), which allowed for easy exchange of the solution isotopic contrast within the (3 mL volume) solid-liquid sample cell. For each isotopic contrast change, a total of 22.5 mL of solution was pumped through the cell (7.5 cell volumes) at a speed of 1.5 mL min^−1^.

Data were processed using standard procedures and fitted using the RasCAL fitting program (version 1, A. Hughes, ISIS Spallation Neutron Source, Rutherford Appleton Laboratory). Details of the fits are provided within the Discussion section.

A single-crystal silicon block (PI-KEM Ltd., Tamworth, UK) measuring 80 mm × 50 mm × 20 mm with a single polished face was first cleaned with a mild Piranha solution (3:1 sulphuric acid: hydrogen peroxide (30%) in 50% H2O) at 80 °C for 30 min before two 30 min rounds of cleaning using a UV ozone cleaner (Ossila Ltd., Sheffield, UK). The polished face was silanized by immersion in a glass Petri dish containing a 0.5% *v*/*v* AAPTMS solution, prepared as above, and incubated for 30 min on a 3D rocking platform (Stuart Scientific, St Neots, UK). The surface was washed with ethanol and sonicated 3 times for 1 min in an ethanol bath before washing with Nanopure water and drying with a nitrogen line. A volume of 15 mL of 20 nm gold nanoparticles, OD525nm = 1, supplemented with 1 mM sodium chloride was added to the silanized face and incubated for 30 min. The solution was agitated every 10 min with a pipette. After 30 min, the nanoparticle solution was removed, and the block was sonicated 3 times for 1 min in a water bath. This process was repeated 3 times.

Protein assembly was carried out within the liquid flow cells by introducing the protein solution via the inlet port and a syringe. Protein samples were prepared by buffer exchange into DDM buffer (0.5% *w*/*v* n-Dodecyl β-D-maltoside, 10 mM Tris HCl pH 8) using a PD10 desalting column (GE Healthcare, Amersham, UK and diluting to 1 mg/mL. The resulting protein solutions were incubated with 25 mM TCEP (Tris(2-carboxyethyl)phosphine hydrochloride) for 30 min to reduce any disulphide bonds and then diluted 1/10 in DDM buffer before use. 1-mercaptoundecyl-11-hexaethylene glycol (filler) solution was prepared by diluting a 50 mM stock 1/100 in DDM buffer supplemented with 25 mM TCEP, incubating for 30 min and then diluting 1/10 in DDM buffer before use.

Glass capillary functionalization. Borosilicate glass capillaries (Hirschmann Laborgeräte GmbH, Eberstadt, Germany) were washed several times with ethanol before use. A 0.5% AAPTMS solution was prepared as described above, then 100 µL was drawn into the capillary and incubated for 30 min. The capillary was rinsed with ethanol and immersed in a beaker of ethanol before sonicating twice for 1 min in an XB2 ultrasonic bath (Grant Instruments, Cambridge, UK). The capillary was then rinsed with Nanopure water before drawing 100 µL of 20 nm gold nanoparticle solution at an OD525nm = 5, then incubated for 30 min. The AuNP solution was removed, and the capillary sonicated twice for 1 min in a beaker of Nanopure water.

The sensitivity of the AuNP functionalized capillaries was measured using a series of glycerol standard solutions made by serial dilution between 30% and 1% (*v/v*). A volume of 2.5 mL of the glycerol standard was injected through the capillary and incubated for 2 min before measuring the UV-Vis spectrum between 400–800 nm. Each measurement was repeated 3 times, and the absorbance of a bare glass capillary filled with Nanopure water was subtracted as a baseline. The barycentric mean wavelength (*λ_m_*), which by integrating over the absorption peak provides an accurate quantification of wavelength shifts, was calculated between 500–600 nm using the equation [41]:

$$\lambda_{m}=\frac{\sum A\left(\lambda \right)\times \lambda }{\sum A\left(\lambda \right)}$$

where *A*(*λ*) is the absorbance at wavelength *λ*.

Protein assembly was carried out in situ in a Cary 4E spectrophotometer (Thermo Fisher Scientific, Horsham, UK) with the capillary inserted into a modified sample stage and connected to a syringe by a section of rubber hose. Protein and filler solutions were prepared as above for the neutron experiments. Firstly, 1 mL of 1% *v*/*v* β-mercaptoethanol was injected and incubated for 5 min before washing with 2 mL of TBS buffer (50 mM Tris HCl pH 8, 150 mM NaCl). Then, 1 mL of protein solution was injected and incubated for 15 min before washing with 2 mL of TBS buffer. A total of 1 mL of 1-mercaptoundecyl-11-hexaethylene glycol solution was then injected and incubated for 15 min. The functionalized capillary was washed with 2 mL of TBS buffer, followed by 1 mL of 1% *w*/*v* SDS solution, and finally rinsed with 2 mL of Nanopure water. A 6 mg/mL stock of recombinant influenza A nucleoprotein (in 20% glycerol storage buffer) was diluted 1 in 100 in Nanopure water prior to injecting 1 mL into the functionalized capillary, then incubated for 5 min before measuring the UV-Vis spectrum between 400–800 nm. Each measurement was repeated 4 times, and the absorbance of a bare glass capillary filled with Nanopure water was subtracted as a baseline. The shift in the barycentric mean (Δλm) was calculated as the difference from the λm of the non-functionalized AuNP surface. The shifts in the barycentric mean for the FluA NP samples were corrected for the change in the refractive index of the bulk solution due to the remaining 0.2% glycerol from the storage buffer when compared with Nanopure water.

## Data Availability

Neutron data are available from the ISIS Pulse Neutron source using the DOI:10.5286/ISIS.E.RB1520380.

## Supplementary Materials

The following supporting information can be downloaded at: https://www.mdpi.com/article/10.3390/ijms24087599/s1.
