# Supplementary material for: Fully Aqueous Self-Assembly of a Gold-Nanoparticle-Based Pathogen Sensor
Source: Int J Mol Sci. 2023 Apr 20;24(8):7599. doi: 10.3390/ijms24087599 (PMC10145400; doi:10.3390/ijms24087599)
Supplement: Supplementary file 1 [file ijms-24-07599-s001.zip › ijms-2322969-supplementary.pdf]

Supplementary data for

Assembly of virus detecting proteins on simple, gold nanoparticle-glass biosensor surfaces

Timothy Robson [1], Deepan S. H. Shah [2], Rebecca J. L. Welbourn [3], Sion R. Phillips [2], Luke A. Clifton [3] and Jeremy H. Lakey [1]

[1] Biosciences Institute, The Medical School, Newcastle University, Framlington Place, Newcastle upon Tyne NE2 4HH, UK

[2] Orla Protein Technologies Ltd., Biosciences Centre, International Centre for Life, Times Square, Newcastle upon Tyne NE1 4EP, UK

[3] SIS Pulsed Neutron and Muon Source, Science and Technology Facilities Council, Rutherford Appleton Laboratory, Harwell Science and Innovation Campus, Didcot, Oxfordshire OX11 OQX, UK

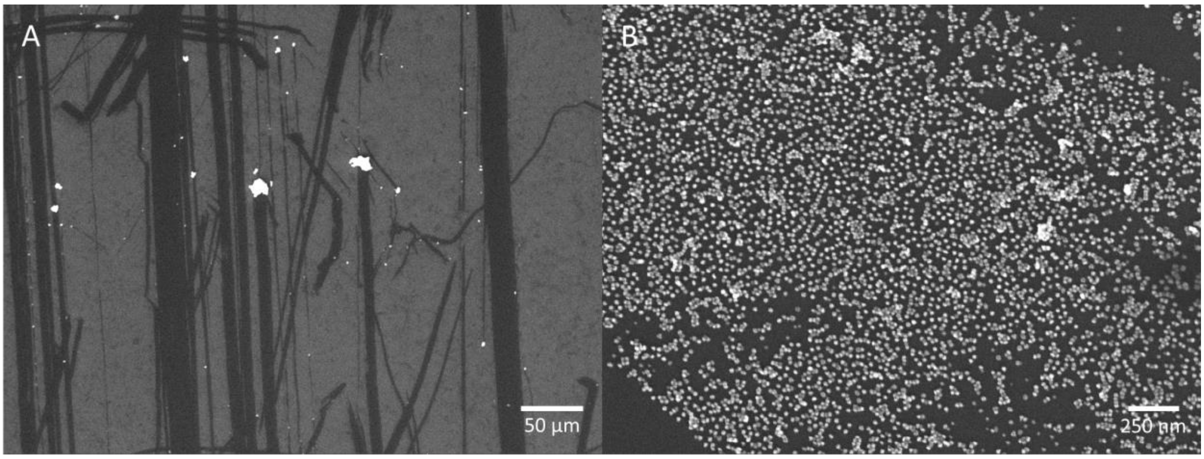

Figure S1 SEM images of the surface of the silicon block after neutron reflectometry experiments with assembled AuNPs and protein arrays. (A) A low magnification (862x) image of the silicon block surface showing significant damage to the assembled AuNPs during transportation from the beamline to the electron microscopy facility. (B) A high magnification image of one of the intact nanoparticle areas demonstrating that a high density of AuNPs remained after protein assembly on the AuNP surface.

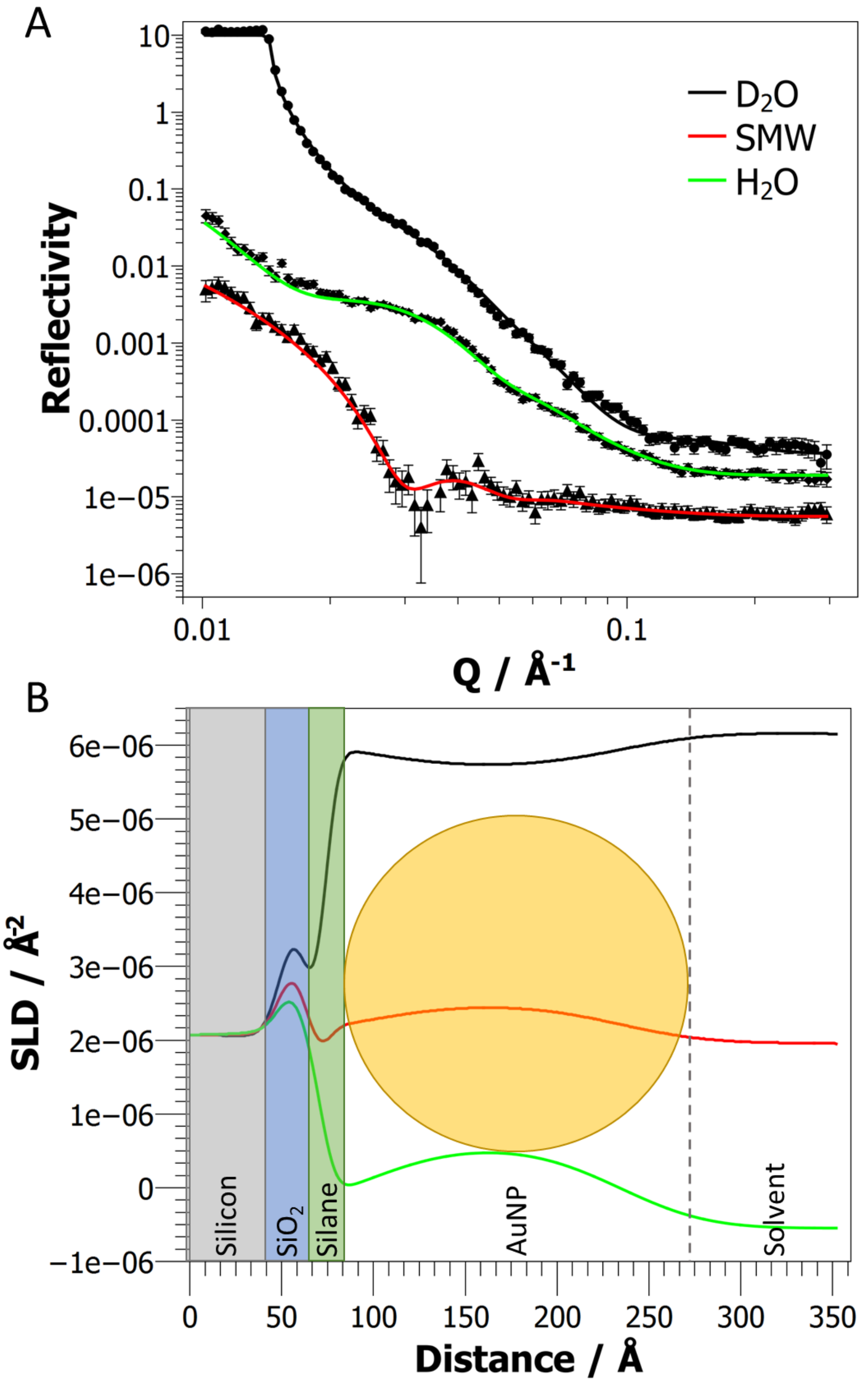

`c

Figure S2 Data fitting and corresponding SLD profiles generated using the slab model for the AuNP coated surface. (A) Reflectivity data with the model fits represented as solid lines and the reflectivity profiles offset for clarity. The colours represent the different solvent contrasts, D_2O, SMW and H_2O. (B) The fitted SLD profiles show the change in the SLD as a function of distance away from the substrate surface, in the Z-Axis. A schematic representation of the surface is overlaid on the SLD profiles to highlight the different regions of the sample. Reflectivity data is available from DOI:10.5286/ISIS.E.RB1520380

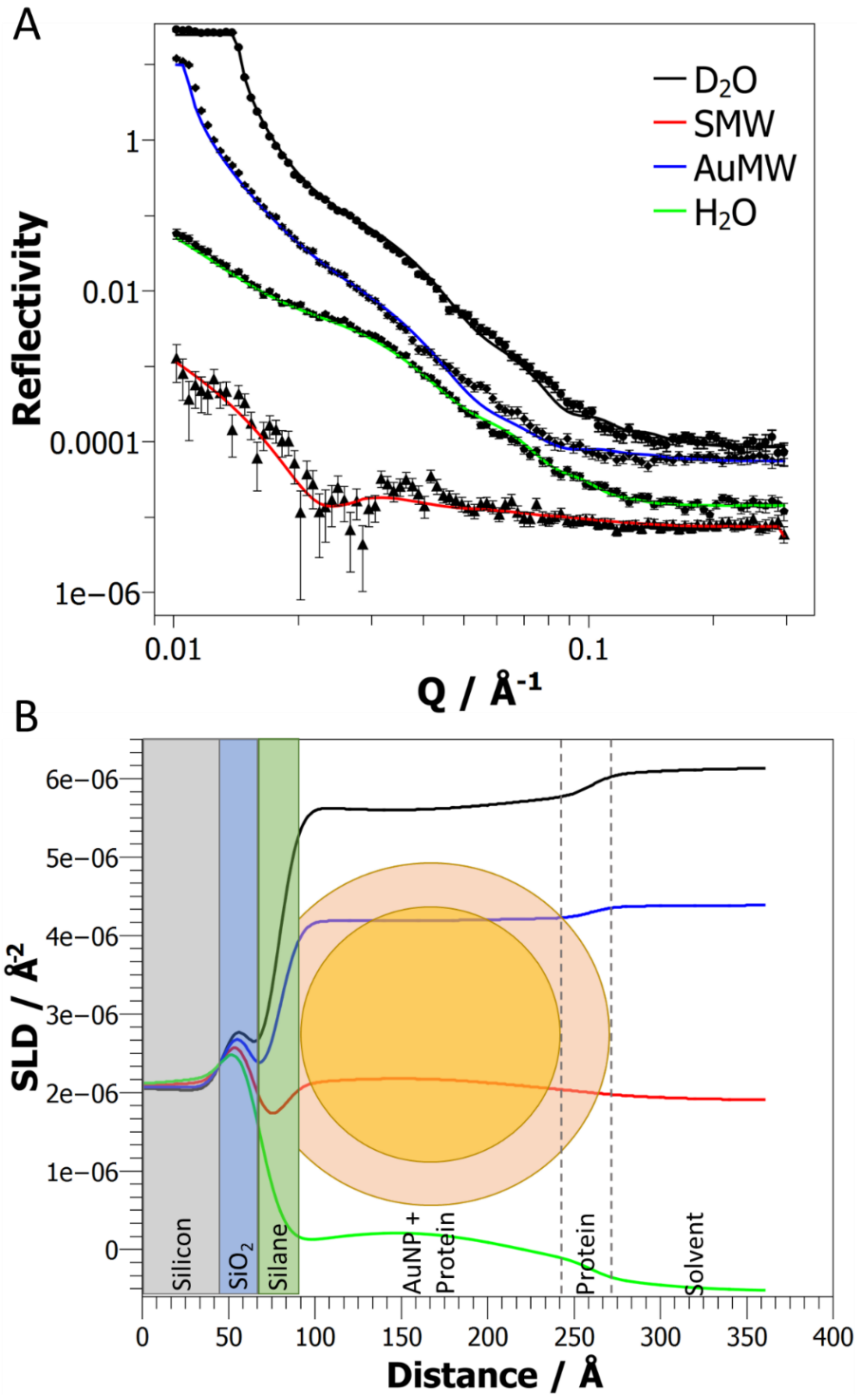

Figure S3 Data fitting and corresponding SLD profiles generated using the slab model for the AuNP coated surface after GGzOmpA$_{TM}$ assembly. (A) Reflectivity data with the model fits represented as solid lines and reflectivity profiles offset for clarity. The colours represent the different solvent contrasts, D_2O, AuMW, SMW and H_2O. (B) A schematic representation of the surface is overlaid on the fitted SLD profiles to highlight the different regions of the sample. The orange layer around the nanoparticle represents the fitted protein and filler layer. Reflectivity data is available from DOI:10.5286/ISIS.E.RB1520380

Table S1 Model parameters fitted for the AuNP surface using the slab model.

PARAMETER	FITTED VALUE
**SUBSTRATE ROUGHNESS / Å**	7.32 ± 1.35
**SIO_2 THICKNESS / Å**	15.04 ± 0.13
**SIO_2 ROUGHNESS / Å**	7.98 ± 0.15
**SIO_2 HYDRATION/ %**	17.47 ± 4.29
**SIO_2 SLD / $Å^{-2}\times10^{-6}$**	3.49 ± 0.06
**SILANE THICKNESS / Å**	8.57 ± 2.00
**SILANE ROUGHNESS / Å**	5.85 ± 2.05
**SILANE HYDRATION / %**	11.04 ± 22.01
**SILANE SLD / $Å^{-2}\times10^{-6}$**	1.00 ± 0.00
**GOLD LAYER THICKNESS/ Å**	179.17 ± 5.65
**GOLD ROUGHNESS / Å**	35.48 ± 10.62
**GOLD COVERAGE / %**	23.01 ± 6.18
**GOLD SLD / $Å^{-2}\times10^{-6}$**	4.18 ± 0.09

Table S2 Model parameters fitted for the GGzOmpA$_{TM}$ coated surface using the slab model.

PARAMETER	FITTED VALUE
**SUBSTRATE ROUGHNESS / Å**	7.93 ± 0.14
**SIO_2 THICKNESS / Å**	14.32 ± 1.27
**SIO_2 ROUGHNESS / Å**	10.50 ± 2.52
**SIO_2 HYDRATION/ %**	12.59 ± 9.42
**SIO_2 SLD / $Å^{-2}$×10^{-6}**	3.48 ± 0.05
**SILANE THICKNESS / Å**	11.92 ± 4.73
**SILANE ROUGHNESS / Å**	10.00 ± 0.1
**SILANE HYDRATION / %**	0.09 ± 18.55
**SILANE SLD / $Å^{-2}$×10^{-6}**	0.57 ± 0.18
**GOLD LAYER THICKNESS/ Å**	153.70 ± 14.25
**GOLD ROUGHNESS / Å**	65.51 ± 15.76
**GOLD COVERAGE / %**	23.71 ± 11.71
**GOLD SLD / $Å^{-2}$×10^{-6}**	4.10 ± 0.00
**PROTEIN THICKNESS / Å**	30.33 ± 9.55
**PROTEIN ROUGHNESS / Å**	9.98 ± 0.05
**PROTEIN SLD / $Å^{-2}$×10^{-6}**	1.49 ± 0.15
**PROTEIN HYDRATION / %**	90.10 ± 0.27

Table S3 Model parameters fitted for the AuNP and GGzOmpA$_{TM}$ coated surfaces using the sphere model.

PARAMETER	FITTED VALUE
**SUBSTRATE ROUGHNESS / Å**	7.69 ± 4.29
**SIO_2 THICKNESS / Å**	15.07 ± 0.15
**SIO_2 ROUGHNESS / Å**	14.44 ± 0.73
**SIO_2 HYDRATION/ %**	45.90 ± 7.03
**SILANE THICKNESS / Å**	8.00 ± 1.00
**SILANE ROUGHNESS / Å**	14.99 ± 0.01
**SILANE HYDRATION / %**	99.80 ± 74.36
**SILANE SLD / $Å^{-2}$×10^{-6}**	0.97 ± 0.15
**GOLD LAYER THICKNESS/ Å**	183.68 ± 4.82
**AUNP COVERAGE (WITHOUT PROTEIN) / %**	38.90 ± 0.78
**AUNP COVERAGE (WITH PROTEIN) / %**	21.44 ± 1.78
**INNER COATING THICKNESS / Å**	22.93 ± 2.93
**INNER COATING HYDRATION / %**	0.05 ± 1.19
**INNER COATING SLD / $Å^{-2}$×10^{-6}**	0.68 ± 0.19
**OUTER COATING THICKNESS / Å**	75.09 ± 0.17
**OUTER COATING HYDRATION / %**	84.40 ± 6.24
